# Investigating altered brain development in infants with congenital heart disease using tensor-based morphometry

**DOI:** 10.1038/s41598-020-72009-3

**Published:** 2020-09-10

**Authors:** Isabel H. X. Ng, Alexandra F. Bonthrone, Christopher J. Kelly, Lucilio Cordero-Grande, Emer J. Hughes, Anthony N. Price, Jana Hutter, Suresh Victor, Andreas Schuh, Daniel Rueckert, Joseph V. Hajnal, John Simpson, A. David Edwards, Mary A. Rutherford, Dafnis Batalle, Serena J. Counsell

**Affiliations:** 1 Centre for the Developing Brain, School of Biomedical Engineering and Imaging Sciences, King’s College London, London, SE1 7EH UK; 2 Biomedical Image Technologies, ETSI Telecomunicación, Universidad Politécnica de Madrid and CIBER-BBN, Madrid, Spain; 3 Biomedical Image Analysis Group, Department of Computing, Imperial College London, London, UK; 4 Biomedical Engineering Department, School of Biomedical Engineering and Imaging Sciences, King’s College London, London, UK; 5 Paediatric Cardiology Department, Evelina London Children’s Hospital, St Thomas’ Hospital, London, UK; 6 Department of Forensic and Neurodevelopmental Science, Institute of Psychiatry, Psychology and Neuroscience, King’s College London, London, UK

**Keywords:** Magnetic resonance imaging, Brain, Congenital heart defects, Paediatric research

## Abstract

Magnetic resonance (MR) imaging studies have demonstrated reduced global and regional brain volumes in infants with congenital heart disease (CHD). This study aimed to provide a more detailed evaluation of altered structural brain development in newborn infants with CHD compared to healthy controls using tensor-based morphometry (TBM). We compared brain development in 64 infants with CHD to 192 age- and sex-matched healthy controls. T2-weighted MR images obtained prior to surgery were analysed to compare voxel-wise differences in structure across the whole brain between groups. Cerebral oxygen delivery (CDO_2_) was measured in infants with CHD (n = 49) using phase contrast MR imaging and the relationship between CDO_2_ and voxel-wise brain structure was assessed using TBM. After correcting for global scaling differences, clusters of significant volume reduction in infants with CHD were demonstrated bilaterally within the basal ganglia, thalami, corpus callosum, occipital, temporal, parietal and frontal lobes, and right hippocampus (p < 0.025 after family-wise error correction). Clusters of significant volume expansion in infants with CHD were identified in cerebrospinal fluid spaces (p < 0.025). After correcting for global brain size, there was no significant association between voxel-wise brain structure and CDO_2_. This study localizes abnormal brain development in infants with CHD, identifying areas of particular vulnerability.

## Introduction

Congenital heart disease (CHD) is the most common congenital abnormality, with a reported prevalence of over 9 per 1,000 live births worldwide^1^. As advances in management of CHD lead to improved mortality rates^2^, morbidity and long-term outcomes have become of increasing concern. Infants and children with CHD may have impaired neurodevelopmental outcomes compared to healthy peers, even in the absence of known genetic syndromes^3–10^. Impaired neurodevelopment in CHD leads not only to short-term impact on early childhood development, but also long-term impact, including poorer school performance and academic achievement, and an increased need for special education services^11–14^.

The neurodevelopmental abnormalities observed in survivors of CHD may be related to abnormal brain development. Magnetic resonance (MR) imaging studies have identified brain injury and abnormal brain development in infants with CHD including impaired cortical folding and white and grey matter microstructural abnormalities^15–26^. In addition, foetuses and infants with CHD have impaired brain growth, including reductions in total and regional brain volumes, compared to healthy controls^22–34^. These abnormalities are evident prior to surgery and have been associated with reduced cerebral oxygen delivery^24,25,33^.

Tensor-based morphometry (TBM) is an MR analysis approach that enables the comparison of variation in shape and volume between the brains of individuals on a voxel-wise basis across the whole brain^35,36^. This method enables increased precision in localizing structural variation between groups compared to methods that involve segmentation of pre-selected brain regions^37^. Of note, TBM is able to identify structural differences that are independent of global scaling differences, ensuring observed differences do not merely reflect differences in global brain volume and head size.

The primary aim of this study was to assess differences in brain structure between infants with CHD and healthy controls using TBM in order to identify those areas at risk of abnormal development in infants with CHD. Our secondary aim was to assess the relationship between structural brain development at a voxel-wise level and cerebral oxygen delivery (CDO_2_) in infants with CHD.

## Results

### Demographics

We studied 64 infants with CHD (35 male) and 192 healthy controls (105 male). There were no significant differences in gestational age (GA) at birth, post-menstrual age (PMA) at scan, sex, birthweight, head circumference at birth, head circumference at scan, or mode of delivery between infants with CHD and healthy controls (Table 1).

**Table 1:** Demographic details of infants with congenital heart disease (CHD) and healthy controls.

Variable	Infants with CHD (n = 64)	Healthy controls (n = 192)	*p*-value
Gestational age at birth, weeks	38.57 (35.29–41.57)	38.86 (35.14–40.43)	0.06^†^
Post-menstrual age at scan, weeks	39.29 (36.43–42.29)	39.43 (36.43–42.86)	0.23^†^
Male, no. (%)	35 (55)	105 (55)	1.00^#^
Birth weight, kg	3.11 (1.81–4.29)	3.11 (1.87–4.80)	0.37*
Birth weight z-score	− 0.83 (− 4.32 to 1.58)	− 0.65 (− 3.57 to 3.12)	0.23*
Birth head circumference, cm	33.70 (29.00–38.50)	34.00 (30.00–37.00)	0.48^†^
Birth head circumference z-score	− 0.60 (− 4.92 to 3.09)	− 0.27 (− 3.96 to 2.75)	0.24^†^
Head circumference at scan, cm	34.00 (29.50–37.40)	34.00 (28.50–37.50)	0.35^†^
Head circumference at scan z-score	− 0.81 (− 4.60 to 2.15)	− 0.48 (− 5.61 to 2.70)	0.30^†^
**Mode of delivery, no. (%)**			
Spontaneous/induced vaginal delivery	24 (38)	58 (30)	0.07^#^
Instrumental delivery	11 (17)	38 (20)	
Elective C-section	7 (11)	48 (25)	
Emergency C-section	22 (34)	48 (25)	
**Cardiac diagnosis, no. (%)**			
Transposition of the great arteries	29 (45)	–	–
Coarctation of the aorta	15 (23)	–	–
Hypoplastic left heart syndrome	3 (5)	–	–
Pulmonary atresia	2 (3)	–	–
Tricuspid atresia	2 (3)	–	–
Tetralogy of Fallot	7 (11)	–	–
Pulmonary stenosis	4 (6)	–	–
Truncus arteriosus	1 (2)	–	–
Large ventricular septal defect	1 (2)	–	–

Values shown are median (range), except where indicated.

*Student’s t-test used.

^†^Mann–Whitney U test used.

^#^Chi-squared test used.

Of the 64 infants with CHD, white matter injury was identified in 19 infants and cerebellar haemorrhage was identified in 4 infants on MR imaging (Supplementary Table S1). Sixty of the 64 infants were diagnosed with CHD antenatally.

### Tensor-based morphometry

#### CHD versus healthy controls

**Figure 1:**
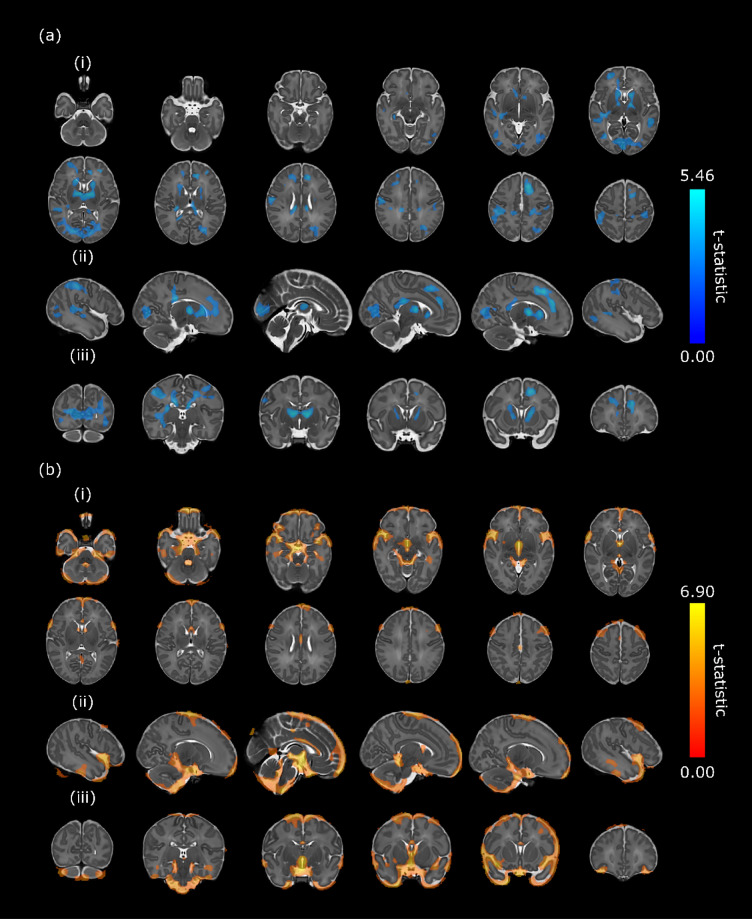
Map of *t*-statistic values of areas of significant **(a)** reductions and **(b)** expansions in volumes in infants with congenital heart disease compared to healthy controls (family-wise-error-corrected *p* < 0.025). Images in 3 planes are shown: (i) axial, (ii) sagittal, (iii) coronal. *t*-statistic range is shown on the colour bars. Results are overlaid on the template image with post-menstrual age at scan of 40 weeks. Left–right orientation follows radiological convention. Sagittal views are presented from right to left.

After factoring out global scaling differences, infants with CHD had clusters of significantly reduced volumes within the deep grey matter, corpus callosum, temporal, parietal, occipital and frontal lobes, compared to controls (Fig. 1a). Specifically, significant reductions in volume were identified bilaterally in the caudate nuclei, globus pallidi and anterior and medial thalami; in the right hippocampus; in the isthmus and splenium of the corpus callosum; the posterior cingulate gyri; within the frontal lobes bilaterally; the postcentral gyri; the medial occipital lobes, around the calcarine fissures and along the optic radiations; bilaterally within the middle and posterior parts of the superior temporal gyri and in the posterior part of the medial and inferior temporal gyri.

There were clusters of significantly expanded volumes in cerebrospinal fluid (CSF) spaces compared to controls (Fig. 1b). Significantly expanded volumes were identified within the cisterna magna, cisterna pontis, and interpenduncular cistern, as well as bilaterally in the ambient cisterns, and extra-axial spaces around the sylvian fissures, frontal lobes, parietal lobes and inferior poles of the temporal lobes. Symmetrical clusters of significant expansions were also found in the third ventricle, fourth ventricle and in the caudothalamic notches bilaterally.

On comparison of total brain and regional brain volumes, we found that infants with CHD had significantly smaller total brain, cortical grey matter, white matter, deep grey matter, and cerebellum volumes than controls (Supplementary Table S2). There was no significant difference in relative regional volumes between groups (Supplementary Table S2). The results of subgroup analysis of abnormal mixing cardiac lesions (n = 31), left-sided cardiac lesions (n = 18), and right-sided cardiac lesions (n = 15) compared to matched controls are included in Supplementary Figure S1.

#### Brain development and cerebral oxygen delivery

Forty-nine infants with CHD underwent phase contrast flow imaging and cerebral blood flow (CBF) was measured and used to calculate CDO_2_. The median CBF was 84.58 mL/min with range 45.58–123.16 mL/min and the median CDO_2_ was 1657 mLO_2_/min with range 1,106–3,023 mLO_2_/min. There were no significant differences between infants in whom CDO_2_ was measured, and those in whom CDO_2_ was not (Supplementary Table S3). No significant association between voxel-wise brain structure and CDO_2_ or CBF was found on TBM analysis.

There were no significant differences in CBF or CDO_2_ between cardiac subgroups (Supplementary Figure S2). A significant positive correlation was observed between CDO_2_ and total brain, cortical grey matter, and deep grey matter volumes (Supplementary Table S4).

## Discussion

This study identified regions of abnormal brain development on preoperative MR imaging in infants with CHD compared to healthy age- and sex-matched controls after taking into account differences in overall brain size between groups. However, after accounting for global scaling, we did not identify any significant associations between CDO_2_ and structural brain development at a voxel-wise level.

Previous studies have shown reductions in total brain volume, regional brain volumes^23–29^ and biometric measurements of brain size^30,31^, and expansions in extra-axial CSF volume^24^ in infants with CHD prior to surgery. Similar to our previous reports in a subsample of this cohort, we observed smaller total brain, white matter, deep grey matter and regional cortical grey matter volumes in infants with CHD compared to controls^24,25^. However, we did not observe any significant differences in relative regional volumes between infants with CHD and controls, suggesting similar volume reductions across all brain regions at this coarse level of analysis. Our study used TBM to extend these analyses in order to identify specific locations of altered brain development in infants with CHD, taking into account global size differences, without the need for a priori definition of regions of interest or image segmentation.

In the analysis of different CHD subgroups, we observed different patterns of abnormal brain development in infants with different cardiac physiologies. However, there were no significant differences in measures of cerebral haemodynamics between cardiac subgroups. It is not clear whether the smaller regions of volume differences in the left- and right-sided lesion groups compared to controls are due to cardiac physiology or the small sample sizes of these 2 groups.

Assessing brain development may be useful in understanding the basis of impaired long-term neurodevelopment in survivors of CHD. Previous studies have demonstrated significant associations between brain volume and impaired neurodevelopment in this high-risk group. Reduced subcortical grey matter and increased CSF volumes in the neonatal period are associated with impaired behavioural state regulation and visual orienting in infants with CHD prior to surgery^10^. In addition, smaller cortical grey matter, white matter, cerebellar^28^, basal ganglia and thalami, and brainstem^38^ volumes in newborns with CHD after surgery are associated with poorer cognitive and language outcome scores^28^ and below-average IQ^38^ in later infancy^28^ and childhood^38^. These relationships persist into adolescence where reduced white matter, cerebellar^39^, and hippocampal volumes^39,40^ are associated with impaired total IQ and other measures of cognitive, motor and executive functions^39,40^.

In this study, we identified significant clusters of reduced volume in regions that are important for cognitive, behavioural and motor function including the caudate nuclei, globus pallidi, thalami, posterior cingulate gyri, frontal lobes, and the hippocampus. The basal ganglia and thalami are associated with cognitive, affective, somatosensory and motor function^41,42^ and reductions in volume within the thalami and basal ganglia have been linked to adverse neurodevelopment in CHD^38^ and prematurity^43,44^. In particular, the anterior thalami, where we localized volume reduction, have been associated with information processing and attention^42^, memory^42,45^ and spatial navigation^45^. The hippocampi play a crucial role in intellectual function and memory^46–48^ and significant correlations have been observed between hippocampal volume and working memory^40^, verbal comprehension^40^, perceptual reasoning^39^ and total IQ in adolescents with CHD^39,40^. In addition, we localized volume reductions to the posterior cingulate gyri, regions which are linked to memory^49,50^, emotional response^51^, attention^52^ and spatial orientation^49^. We also observed clusters of significant reductions in the frontal lobes. The frontal lobes are associated with many higher cognitive functions, including attention, executive function, impulse control, language, and memory^53^, functions which may be impaired in children with CHD^54–56^.

We localized significant volume expansions in CSF spaces, including the basal cisterns, third and fourth ventricles and caudothalamic notches bilaterally. Increased CSF volume has been found in children with specific language impairment compared to controls^57^ and has been linked to autism spectrum disorder in infants at high genetic risk^58^, and to moderate-severe neurodevelopmental disability^59^ and decreased working memory performance^60^ in preterm infants.

There are a number of potential mechanisms underlying abnormal brain development in infants with CHD including altered cerebral haemodynamics^24,61–63^, reduced substrate delivery to the brain^64^, genetic factors^65–67^ and impaired placental development^68,69^. We previously reported a correlation between CDO_2_ and preoperative neonatal total brain and cortical grey matter volumes in our CHD cohort^24^. In this study, we also observed significant associations between CDO_2_ and total brain, cortical grey matter, and deep grey matter volumes. However, after controlling for global scaling, we did not identify additional clusters of altered development on a voxel-wise level associated with CDO_2_. This suggests that either (i) reduced CDO_2_ impairs growth across the whole brain and there are no regions that are specifically vulnerable to low CDO_2_ or (ii) there was insufficient statistical power to assess voxel-wise differences after correcting for multiple comparisons. Of note, the measurement of CDO_2_ at a single postnatal timepoint may not reflect the full burden of hypoxia experienced by these infants. Indeed, reduced cerebral oxygenation has been associated with reduced brain volume in utero^33^. While the reduction of CDO_2_ is probably global, intrinsic vulnerabilities, such as high metabolic demand, could play a role in the impact on specific brain structures. In other at-risk populations of neonates, such as in those experiencing acute hypoxic-ischaemic events, patterns of injury involving structures with higher metabolic demand, including the basal ganglia and thalami, and hippocampus, have been observed^70,71^.

However, in this study, we observed clusters of volume reductions, in regions including both the grey and white matter. This is presumably because the effect of chronic hypoxia differs from that of acute events, and different regions of the brain have varying metabolic demands at different stages of development^72,73^. Furthermore, cerebral autoregulatory mechanisms play an important role^61^, and different regions of the brain may be affected by different degrees of “brain sparing”^74^, particularly as the cerebral vasculature is itself developing during mid-late gestation^75^.

A limitation of this study is that, as yet, we do not have neurodevelopmental outcome data for all of the infants. Further studies are required to examine the relationship between regions of altered brain development identified on voxel-wise analyses and neurodevelopmental outcome.

## Conclusions

Using tensor-based morphometry, this study identified clusters of localized volume alterations in regions important for subsequent cognitive, behavioural and motor function in infants with CHD compared to healthy control infants. Whilst cerebral oxygen delivery was significantly associated with total brain and regional brain volumes, no significant relationship was found between cerebral oxygen delivery and voxel-wise brain volume after controlling for global size differences. Assessing brain structural abnormalities using a voxel-wise approach, such as tensor-based morphometry, refines our understanding of abnormal brain development in infants with CHD.

## Methods

The project was approved by the National Research Ethics Service West London committee (CHD: 07/H0707/105; Controls: 14/LO/1169). Informed written parental consent was obtained before imaging. All methods and experiments were carried out in compliance with relevant guidelines and regulations.

### Participants

**Figure 2:**
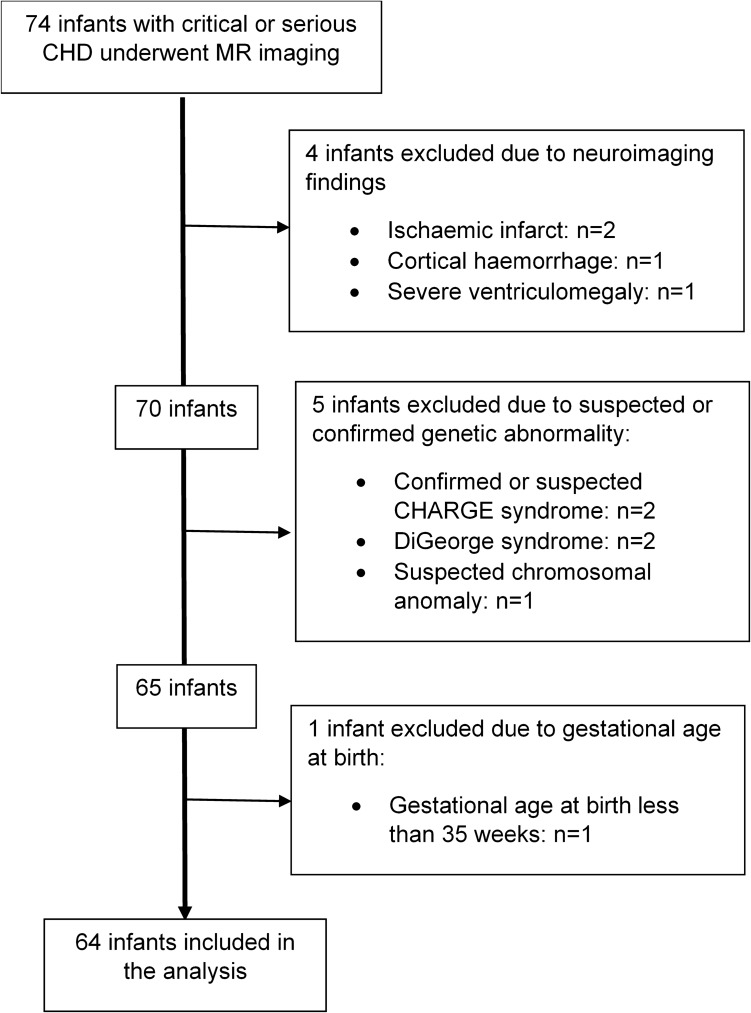
Flowchart showing number of cases excluded from further analysis.

A prospective cohort of 74 infants born with critical or serious CHD requiring surgery within one year, based on previously published UK classification^76,77^, was recruited for MR neuroimaging from the Neonatal Intensive Care Unit at St Thomas’ Hospital, London. Exclusion criteria were known or suspected genetic syndromes, major abnormalities on MR imaging and GA at birth < 35 weeks. Sixty-four infants were included in the analysis (Fig. 2). Healthy controls were obtained from the Developing Human Connectome Project (dHCP) database^78^ and were matched for GA at birth, PMA at scan, and sex, to each infant in the CHD group in a ratio of three healthy controls per one infant with CHD. Matching of healthy controls to infants with CHD was carried out in R, version 3.2.3^79^, using an automated method based on the daisy dissimilarity matrix calculation^25,80^.

### MR imaging

High-resolution MR imaging was performed on a Philips Achieva 3-T system (Best, The Netherlands) using a 32-channel neonatal head coil and neonatal positioning device^81^. T2-weighted, T1-weighted, and diffusion-weighted MR images were acquired using the dHCP protocol optimized for neonatal scanning^78,81,82^. Phase contrast flow imaging was also acquired for the CHD cases^24^.

T2-weighted images were used for TBM analysis and were acquired using a multi-slice turbo spin echo sequence in 2 stacks of 2-dimensional slices in sagittal and axial planes, with pulse repetition time 12 s, echo time 156 ms, flip angle 90°, slice thickness 1.6 mm with 0.8 mm overlap, in-plane resolution 0.8 mm × 0.8 mm. Quantitative flow imaging was acquired using velocity-sensitized phase contrast imaging, with a single-slice spoiled gradient echo sequence, with field of view 100 mm × 100 mm, acquisition resolution 0.6 mm × 0.6 mm × 4.0 mm, repetition time 6.4 ms, echo time 4.3 ms, flip angle 10°, 20 cardiac phases, maximal encoding velocity 140 cm/s, and scan time 71 s^83^. Other imaging parameters have been previously described^25^. Infants were scanned in natural sleep without sedation, in the presence of a paediatrician experienced in MR imaging procedures. All images were reviewed for abnormalities by a paediatric neuroradiologist.

Infants had physiological monitoring (electrocardiography, respiratory rate, oxygen saturations and temperature) throughout the scan. Ear protection was used to minimize impact of scanner noise: ear plugs moulded from putty (President Putty, Coltene Whaledent, Mahwah, NJ, USA) placed in the external auditory meatus and covered with neonatal earmuffs (MiniMuffs, Natus Medical Inc., San Carlos, CA, USA), with an acoustic foam hood for noise absorption positioned over the infant in the scanner.

### Structural image processing

Motion-correction and slice-to-volume image reconstruction were carried out retrospectively using a dedicated algorithm to obtain 0.8 mm^3^ isotropic T2-weighted images^84,85^. These were segmented into tissue type (white matter, grey matter, cerebrospinal fluid (CSF), cerebellum, deep grey matter) using a multi-structure expectation–maximization-based segmentation technique in a neonatal-specific automated pipeline described previously^86–88^.

### Cerebral oxygen delivery

For infants with CHD, cerebral blood flow (CBF) was calculated from phase contrast MR imaging acquired in a plane perpendicular to both internal carotids and basilar arteries at the level of the sphenoid bone, using a previously described method^24,83^. Haemoglobin (Hb) measurements were performed as part of routine clinical care, at a median (range) of 2 (− 1 to 10) days before the scan. Arterial oxygen saturation (SaO_2_) was measured at the time of scan using a Masimo Radical-7 monitor (Masimo Corp, Irvine, CA) applied to the right hand. Cerebral oxygen delivery (CDO_2_) was calculated using the following formula^89^:

$${\text{CDO}}_{{2}} ({\text{mL O}}_{{2}} /{\min}) \, = {\text{ SaO}}_{{2}} \times \, \left[ {{\text{Hb}}} \right] \, \left( {{\text{g}}/{\text{dL}}} \right) \, \times { 1}.{36 } \times \, \left[ {{\text{CBF}}} \right] \, \left( {{\text{mL}}/{\min}} \right)$$

where 1.36 is the amount of oxygen bound per gram of haemoglobin at 1 atmosphere (Hüfner’s constant)^29^.

### Tensor-based morphometry

Neonatal templates constructed for each week of gestation created from the dHCP neuroimaging database were used as templates for registration^90^. Subject T2-weighted images were registered to an age-at-scan-matched template image using the Symmetric Normalization (SyN) algorithm from Advanced Normalization Tools (ANTs), version 3.0^91^. Segmented T2-weighted images were included in the algorithm to improve the quality of registration.

Following rigid and affine transformations of the image, the non-linear transformations from the SyN algorithm were used to create deformation tensor fields within the template space. By using only non-linear transformations, global volume differences are factored out. The resulting tensor fields describing the voxel-wise shape and volume change from the template to each subject image were used to calculate scalar Jacobian determinants, which were subject to logarithm transformation, using ANTs^92^.

Log-Jacobian determinant maps were smoothed with a sigma of 3.5 mm full width at half maximum Gaussian filter. To facilitate processing, the log-Jacobian determinant maps were re-sized to a voxel size of 1 mm^3^ isotropic prior to statistical analysis.

### Statistical analysis

Clinical data were compared between groups using Student’s *t*-test or Mann–Whitney U for continuous variables, with prior normality testing using the Shapiro–Wilk test. Chi-squared test was used for categorical variables. A *p*-value of less than 0.05 was taken as significant. Missing values for clinical data were dealt with through pairwise deletion. Statistical analysis was performed with IBM SPSS Statistics for Windows, version 24 (IBM Corp., Armonk, N.Y., USA). Z-scores for birth weight and head circumference were calculated using the GrowthCharts^UK-WHO^ application, version 2.0.1^93^, based on UK-WHO 2006 population reference data^94^.

For TBM analysis, the 64 infants with CHD were first compared to age- and sex-matched healthy controls. Voxel-wise *t*-tests of log-Jacobian determinants between CHD and control groups were carried out using FSL Randomise (FSL, version 6.01)^95,96^. Threshold-free cluster enhancement was used with a random permutation method with 10 000 permutations, based on a General Linear Model (GLM) matrix^95,97^. Additionally, for CHD infants with successful phase contrast flow imaging (n = 49), voxel-wise regression analyses of log-Jacobian determinants with CDO_2_ and CBF were performed. A brain mask for the template image at a PMA of 40-weeks was used in all analyses to include only brain tissue and CSF in the comparisons^90^. GA at birth and sex were included as nuisance variables in each model. *P*-values were corrected for multiple comparisons. A family-wise-error-corrected *p*-value of less than 0.025 was considered significant, to account for testing differences in two directions (i.e. CHD > controls and CHD < controls, or in the case of CDO_2_, a positive and negative correlation with CDO_2_).

Further supplementary methods are included within the supplementary information.

## Supplementary information

Supplementary Information.

## Data Availability

Anonymised data pertaining to the Developing Human Connectome Project have been made publicly available at the Developing Human Connectome Project repository and can be accessed at https://www.developingconnectome.org/. The remaining data, analytic methods, and study materials will be available to other researchers for the purposes of reproducing the results or replicating the procedure on reasonable request.
