# Supplementary material for: Investigating altered brain development in infants with congenital heart disease using tensor-based morphometry
Source: Sci Rep. 2020 Sep 10;10:14909. doi: 10.1038/s41598-020-72009-3 (PMC7483731; doi:10.1038/s41598-020-72009-3)
Supplement: Supplementary file 1 — Supplementary Information. [file 41598_2020_72009_MOESM1_ESM.docx]

**Supplementary information**

**Investigating altered brain development in infants with congenital heart disease using tensor-based morphometry.**

Isabel H. X. Ng, Alexandra F. Bonthrone, Christopher J. Kelly, Lucilio Cordero-Grande, Emer J. Hughes, Anthony N. Price, Jana Hutter, Suresh Victor, Andreas Schuh, Daniel Rueckert, Joseph V. Hajnal, John Simpson, A. David Edwards, Mary A. Rutherford, Dafnis Batalle, Serena J. Counsell.

**Supplementary Methods**

**Brain injury in infants with congenital heart disease (CHD)**

All magnetic resonance images were reviewed and reported by a paediatric neuroradiologist. In infants with white matter lesions, injury was classified into mild (0 – 3 foci and all ≤2mm), moderate (4 – 10 foci or any >2mm), or severe (>10 foci)^[76, 98]^.

**CHD subgroup analysis**

For the CHD subgroup analysis, the 64 infants with CHD were classified into 3 subgroups based on cardiac physiology: abnormal mixing lesions (transposition of the great arteries, truncus arteriosus, ventricular septal defect), left-sided lesions (coarctation of the aorta, hypoplastic left heart syndrome), or right-sided lesions (pulmonary stenosis, pulmonary atresia, tetralogy of Fallot, tricuspid atresia). Each subgroup was compared to their age- and sex-matched healthy controls in 3 separate tensor-based morphometry analyses. Methods for tensor-based morphometry are as outlined in the main manuscript for whole-group analysis.

**Statistical analyses**

Total brain and regional volumes of different tissue types were compared between infants with CHD and healthy controls. Relative regional volumes, each tissue type volume as a ratio of total brain volume, were also compared. General linear models were used with analysis of covariance (ANCOVA), with post-menstrual age at scan and sex as co-variates, to compare values between groups.

When comparing demographic variables between groups, continuous variables were compared using Student’s t-test or Mann-Whitney U, with prior normality testing using the Shapiro-Wilk test. Chi-squared test was used for categorical variables. The Kruskal-Wallis test was used to compare the measures of cerebral blood flow (CBF) and cerebral oxygen delivery (CDO_2_) between cardiac subgroups. Linear regression analyses were carried out to compare the relationship between cerebral oxygen delivery (CDO_2_) and total brain and regional brain volumes.

**Supplementary Results**

**Brain injury in infants with congenital heart disease**

| **Type of brain injury** | **Number of infants (%)** |
| --- | --- |
| White matter injury | 19 (30) |
| - Mild | 13 (20) |
| - Moderate | 5 (8) |
| - Severe | 1 (2) |
| Cerebellar haemorrhage | 4 (6) |

**Supplementary Table S1:** Table displaying the number (percentage) of infants with congenital heart disease included in the analysis who had evidence of brain injury on magnetic resonance imaging.

**Volume differences between infants with congenital heart disease (CHD) and healthy controls.**

Infants with CHD had significantly smaller total brain volume and regional volumes of cortical grey matter, white matter, cerebellum and deep grey matter. There were no significant differences in relative regional volumes between groups.

| **Region** | **Volume, mL** | | **p-value** | **Relative volume** | | **p-value** |
| --- | --- | --- | --- | --- | --- | --- |
|  | **CHD (n=64)** | **Controls (n=192)** |  | **CHD (n=64)** | **Controls (n=192)** |  |
| Total brain | 312.839 (32.940) | 333.688 (40.327) | <0.0005^*^ | – | | |
| Cortical grey matter | 123.551 (15.298) | 132.984 (21.089) | <0.0005^*^ | 0.394 (0.014) | 0.397 (0.021) | 0.717 |
| White matter | 136.635 (14.450) | 145.093 (15.164) | <0.0005^*^ | 0.437 (0.016) | 0.436 (0.022) | 0.496 |
| Cerebellum | 20.804 (2.673) | 22.015 (3.401) | 0.002^*^ | 0.067 (0.005) | 0.066 (0.005) | 0.186 |
| Deep grey matter | 23.699 (2.155) | 25.083 (2.630) | <0.0005^*^ | 0.076 (0.003) | 0.075 (0.004) | 0.408 |

**Supplementary Table S2:** Comparison of absolute and relative volumes between infants with congenital heart disease and healthy controls. Means (standard deviation) are displayed. Comparison between groups was performed using general linear models and analysis of covariance tests with post-menstrual age at scan and sex as co-variates. Bonferroni correction leads to a significance threshold of 0.01. ^*^ - significant association.

**Differences in brain structure between infants with different cardiac physiologies compared to controls**

The largest subgroup was infants with abnormal mixing lesions (n=31). When we compared these cases to their matched controls (n=93), we observed a similar pattern to the whole group analysis with significant reductions in volume bilaterally in regions of the caudate nuclei, globus pallidi, anterior and medial thalami, and anterosuperior frontal lobes, and unilaterally in the left inferior frontal lobe, left superior temporal gyrus and left optic radiation. Significant expansions in volume were observed in the cerebrospinal fluid spaces (Supplementary Figure S1a).

Infants with left-sided lesions (n=18) versus matched healthy controls (n=54) demonstrated significant reductions in volume in the medial occipital lobes around the calcarine fissures and significantly expanded areas in the region of the superior cerebellar cisterns bilaterally (Supplementary Figure S1b).

In the comparison between infants with right-sided lesions (n=15) and their matched healthy controls (n=45), only one single cluster of significant expansion in volume was found in the right extra-axial space above the parietal lobe (Supplementary Figure S1c).


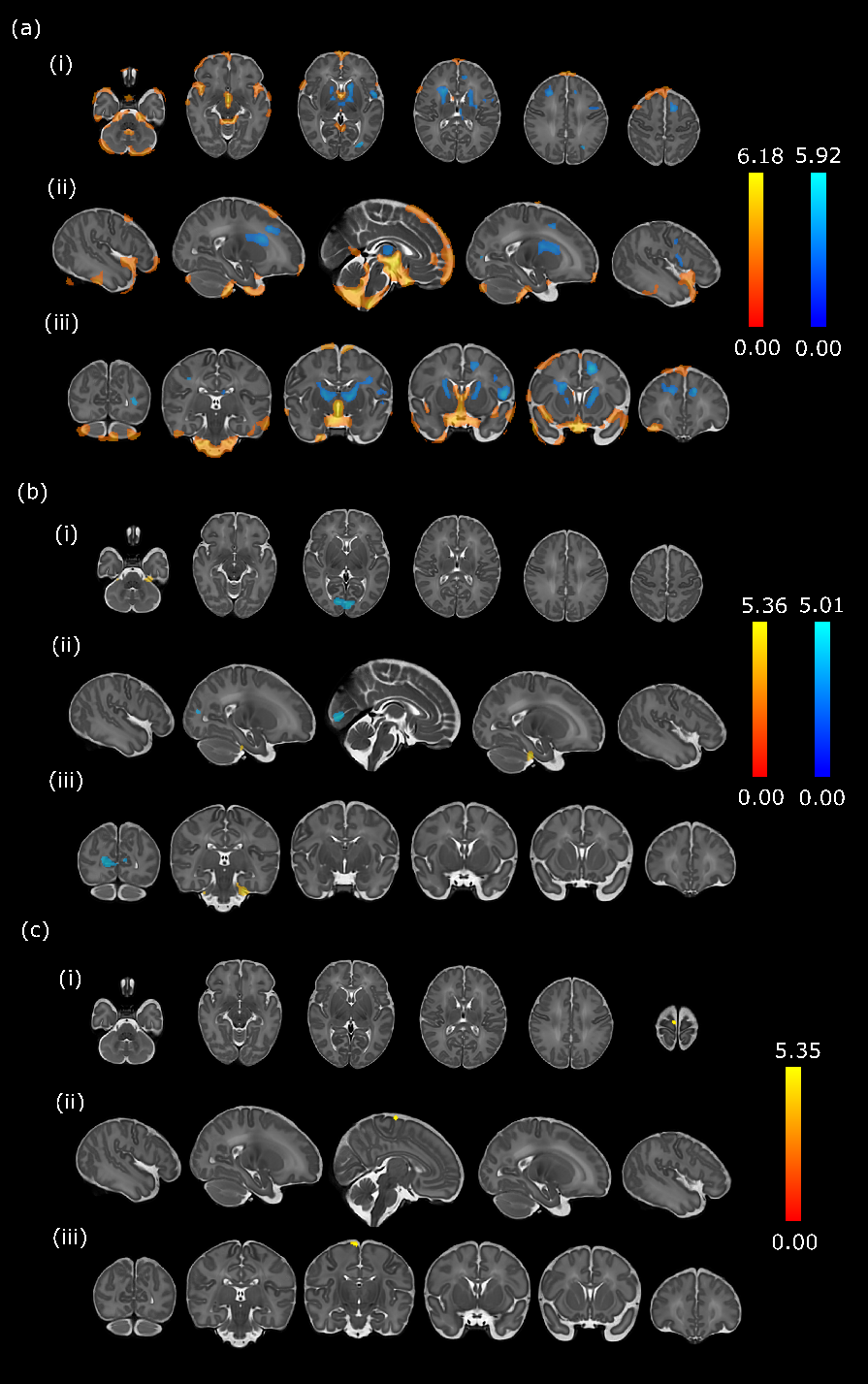


**Supplementary Figure S1:** Map of t-statistic values of areas of significant volume differences in subgroup analysis in infants with (a) abnormal mixing cardiac lesions, (b) left-sided cardiac lesions, and (c) right-sided cardiac lesions. Images in 3 planes are shown: (i) axial, (ii) sagittal, (iii) coronal. t-statistic range is shown on colour bars. Significantly expanded volumes are coloured in red – yellow; significantly reduced volumes are coloured in blue – light-blue. Results are overlaid on the template image with post-menstrual age at scan of 40 weeks. Left-right orientation follows radiological convention. Sagittal views are presented from right to left.

**Demographic details of infants with congenital heart disease (CHD) included in the analysis of cerebral oxygen delivery (CDO_2_) and those that were not.**

Of the 64 infants with CHD, 8 infants did not have phase contrast flow imaging, 7 infants underwent phase contrast flow imaging but had unmeasurable cerebral blood flow (CBF) such that CDO_2_ could not be calculated, and 49 infants underwent phase contrast flow imaging and had CDO_2_ calculated.

There were no significant differences in demographic details between infants who had CDO_2_ calculated and those that did not.

| **Variable** | **CDO_2_ calculated (n=49)** | **CDO_2_ not calculated (n=15)** | ***p*-value** |
| --- | --- | --- | --- |
| Gestational age at birth, weeks | 38.57 (35.29 – 41.57) | 38.43 (36.43 – 41.43) | 0.51^†^ |
| Post-menstrual age at scan, weeks | 39.29 (36.43 – 41.86) | 39.14 (38.14 – 42.29) | 0.50^†^ |
| Male, no. (%) | 28 (57) | 7 (47) | 0.48^#^ |
| Birth weight, kg | 3.11 (1.81 – 4.29) | 3.10 (2.12 – 3.63) | 0.80^*^ |
| Birth weight z-score | -0.83 (-4.32 – 1.58) | -0.75 (-3.19 – 0.50) | 0.83^*^ |
| Birth head circumference, cm | 34.00 (29.00 – 38.50) | 33.60 (29.30 – 35.60) | 0.49^*^ |
| Birth head circumference z-score | -0.60 (-4.92 – 3.09) | -1.08 (-2.70 – 1.41) | 0.53^*^ |
| Head circumference at scan, cm | 34.00 (29.50 – 37.40) | 34.50 (31.30 – 35.60) | 0.93^*^ |
| Head circumference at scan z-score | -0.87 (-4.60 – 2.15) | -0.20 (-2.81 – 1.41) | 0.65^*^ |
| Mode of delivery, no. (%) | | | |
| - Spontaneous/induced vaginal delivery | 18 (37) | 6 (40) | 0.57^#^ |
| - Instrumental delivery | 9 (18) | 2 (13) |  |
| - Elective C-section | 4 (8) | 3 (20) |  |
| - Emergency C-section | 18 (37) | 4 (27) |  |
| Cardiac subgroup | | | |
| - Abnormal mixing lesions | 25 (51) | 6 (40) | 0.50^#^ |
| - Left-sided lesions | 12 (24) | 6 (40) |  |
| - Right-sided lesions | 12 (24) | 3 (20) |  |

**Supplementary Table S3:** Table displaying demographic details of infants with CHD that did compared to those that did not have CDO_2_ successfully calculated from phase contrast imaging. Values shown are median (range), except where indicated. * Student’s t-test used, † Mann-Whitney U test used, # Chi-squared test used.

**Cerebral haemodynamics in different cardiac subgroups**

There were no significant differences in cerebral blood flow or cerebral oxygen delivery between CHD groups.

**
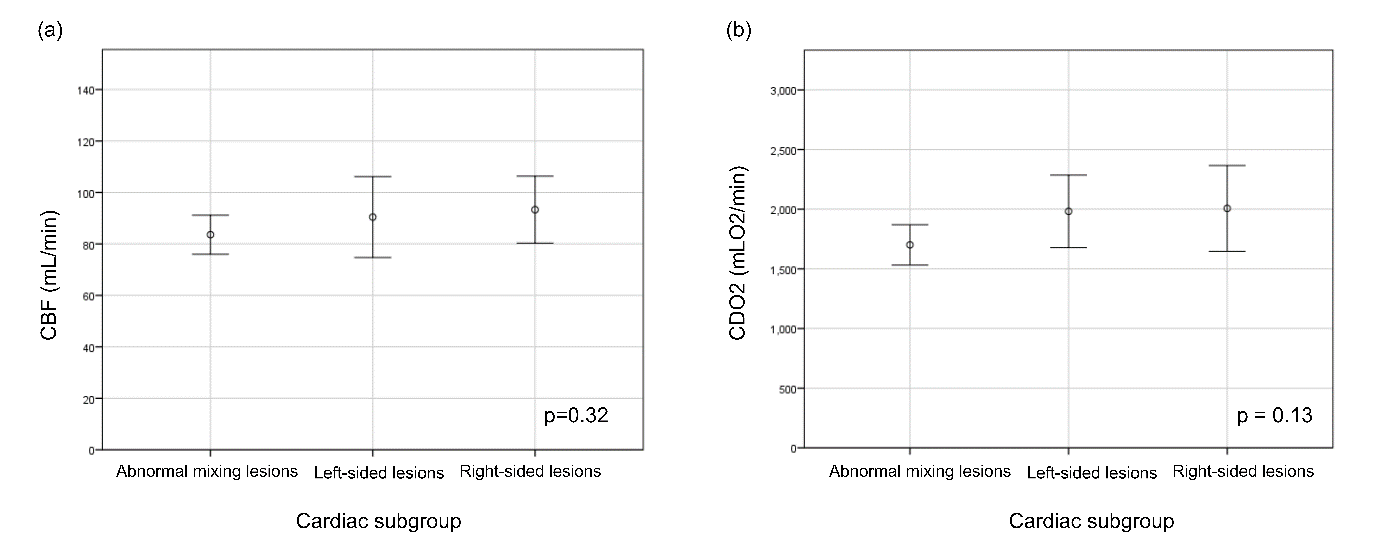
Supplementary Figure S2:** Comparing (a) cerebral blood flow and (b) cerebral oxygen delivery between infants grouped by cardiac physiology. Means and 95% confidence intervals are shown. Kruskal-Wallis test was used to compare cerebral blood flow and cerebral oxygen delivery between the three groups.

**The association between cerebral oxygen delivery and total and regional brain volumes.**

Significant positive correlations were found between CDO_2_ and total brain, cortical grey matter and deep grey matter volumes.

| **Volume** | **R^2^** | **B coefficient** | **p-value** |
| --- | --- | --- | --- |
| Total brain | 0.155 | 29.096 | 0.005^*^ |
| Cortical grey matter | 0.158 | 13.533 | 0.005^*^ |
| White matter | 0.115 | 11.040 | 0.017 |
| Cerebellum | 0.129 | 2.033 | 0.011 |
| Deep grey matter | 0.172 | 1.960 | 0.003^*^ |

**Supplementary Table S4:** Regression analyses comparing brain volumes to cerebral oxygen delivery. Bonferroni correction leads to a significance threshold of 0.01. ^*^: significant association.

**Labelled areas of significant reductions in brain volume in infants with congenital heart disease (n=64) compared to matched controls (n=192)**

**
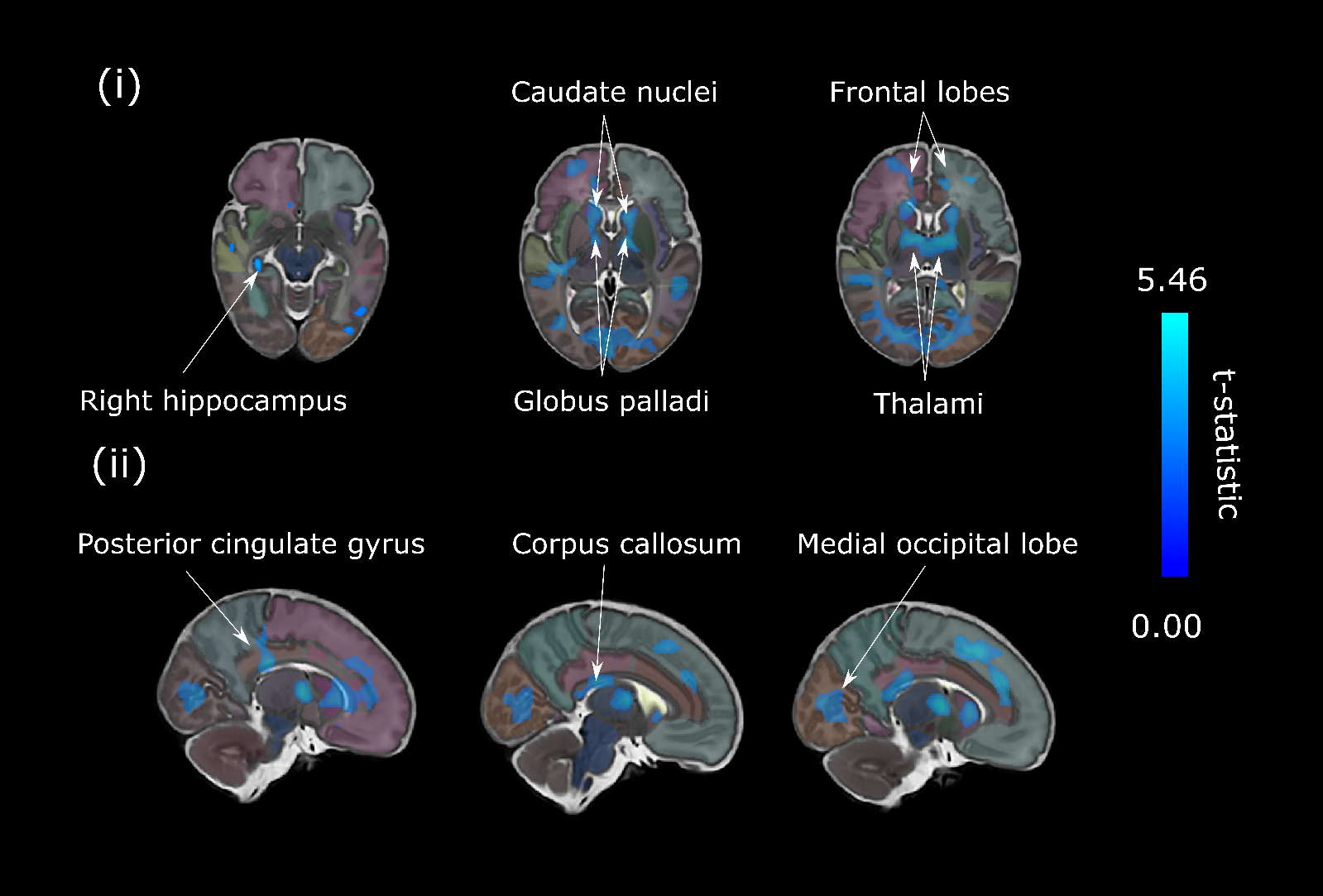
**

**Supplementary Figure S3:** Map of t-statistic values of areas of significant reductions in brain volume in infants with congenital heart disease compared to healthy controls (family-wise-error-corrected p<0.025). Images in 2 planes are shown: (i) axial, (ii) sagittal. t-statistic range is shown on the colour bars. Results are overlaid on the template image with post-menstrual age at scan of 40 weeks. Left-right orientation follows radiological convention. Sagittal views are presented from right to left.
